# Supplementary material for: PEAβ Triggers Cognitive Decline and Amyloid Burden in a Novel Mouse Model of Alzheimer’s Disease
Source: Int J Mol Sci. 2021 Jun 30;22(13):7062. doi: 10.3390/ijms22137062 (PMC8267711; doi:10.3390/ijms22137062)
Supplement: Supplementary file 1 [file ijms-22-07062-s001.zip › ijms-1262282-supplementary.pdf]

Supplementary Tables and Figures

Supplementary Table S1: Premature death of mice from TAPS and parental lines. All animals within this analysis are littermates derived from the same breedings.

		**Premature death**	
**Genotype**	Total number	number	%
**TAPS**	37	11	30
**WT**	67	11	16
**APP/PS1**	42	8	19
**TBA2.1**	50	7	14

Supplementary Table S2: Means of body weights by gender, genotype and age. Data is given in grams +/- SEM, showing no significant differences between genotypes of same gender and age over time.

**Body Weight (g)**							
		**Age (months)**					
**Gender**	**Genotype**	4	6	9	12	15	18
Male	TAPS	32.3 ± 1.2	28.6 ± 2.2	36.0 ± 1.7	35.8 ± 1.6	36.8 ±1.7	38.0 ± 2.7
	WT	36.8 ± 6.2	28.5 ± 2.2	34.7 ± 2.1	37.8 ± 3.0	37.6 ± 4.0	34.7 ± 3.6
Female	TAPS	24.0	26.0 ± 0.8	28.6 ± 1.5	29.9 ±1.9	40.0 ±0.8	-
	WT	22.5	32.0	30.3 ± 3.0	30.8 ±2.8	33.0 ± 2.9	-

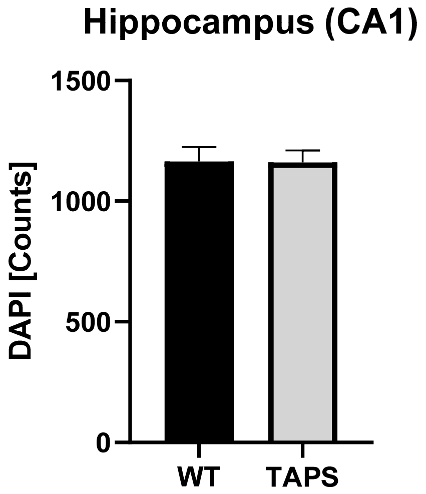

Supplementary Figure S1: Neuronal quantification in the CA1 region of the hippocampus of TAPS and wild type (WT) mice. The number of neurons in the stratum pyramidale was quantified after DAPI staining of cell nuclei. TAPS mice had similar amount of neurons in the CA1 region of the hippocampus compared to WT. Data is given as mean + SEM; * $p < 0.05$; ** $p < 0.01$; *** $p < 0.001$; **** $p < 0.0001$.

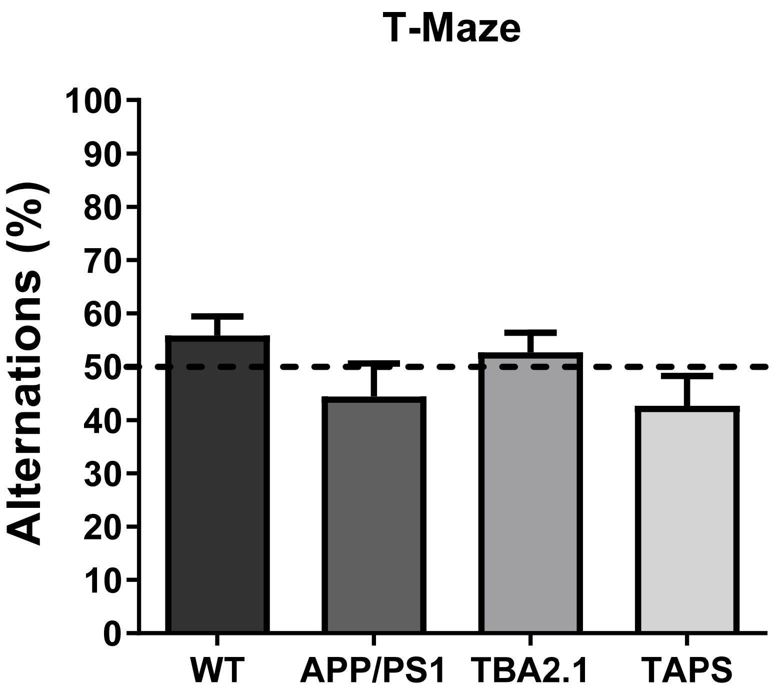

Supplementary Figure S2: Similar performance of all mice in the T-maze at 18 months. The spontaneous alternation was calculated as ratio of entries into the correct arm to the amount of total trials in the T-maze. With 18 months of age, the APP/PS1, TAPS and TBA2.1 mice

alternated similarly compared to the wild type (WT) mice. None of the groups alternated significantly more than 50%, which it the chance level of choosing the correct arm (dashed line). Data is given as mean + SEM.

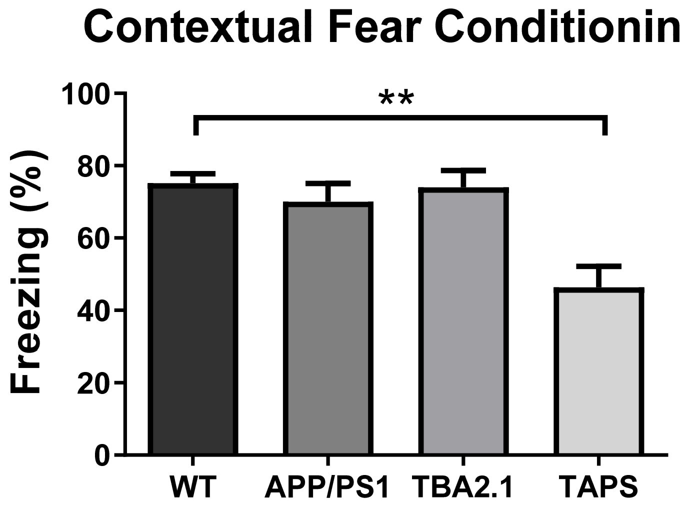

Supplementary Figure S3: Impaired fear conditioning learning in TAPS mice. The percentage of freezing behavior was measured in the Contextual Fear Conditioning paradigm with 20 months of age. In the Contextual Fear Conditioning, the TAPS mice froze less compared to the wild type (WT) indicating a deficit in fear conditioning learning (** $p = 0.0042$). Both APP/PS1 and TBA2.1 showed similar freezing behavior compared to WT, indicating intact fear conditioning learning. Data is given as mean + SEM.
